# Evaluating the Benefits of Viral Respiratory Panel Test in the Reduction of Emergency Department Throughput Time for Patients With Acute Exacerbation of Chronic Obstructive Pulmonary Disease

**DOI:** 10.7759/cureus.19213

**Published:** 2021-11-02

**Authors:** Nnennaya U Opara, Brian M Hensley, Caleb Judy

**Affiliations:** 1 Emergency Medicine, Charleston Area Medical Center (CAMC) Education and Research Institute, Charleston, USA; 2 Emergency Medicine, Charleston Area Medical Center, Charleston, USA

**Keywords:** viral respiratory panel, acute exacerbation of chronic obstructive pulmonary disease, throughput time, emergency department, cough

## Abstract

Introduction

There has been a widespread antibiotic prescription in the Emergency Department (ED) among patients presenting with acute exacerbation chronic obstructive pulmonary disease (AECOPD) irrespective of the causative agent of the disease. The viral respiratory panel (VRP) test is designed to detect viral pathogens in the respiratory tract, which may contribute to the exacerbation of chronic obstructive pulmonary disease (COPD), as the upper and lower respiratory tract infections are caused by a broad range of microbes and not only bacteria. The aim of this study is to weigh the benefits of obtaining a VRP in patients presenting with isolated symptoms pertaining to well-defined criteria of an AECOPD with preexisting COPD or reactive airway disease to find out how such test impacts patient throughput time in the ED and also investigate how obtaining a VRP affects the use of antibiotics in this patient population. It is important that ED physicians accurately diagnose the main cause of AECOPD to help optimize the use of health care resources, including antibiotics, antivirals, inpatient, and ED beds. VRP testing must be taken into consideration as it helps eliminate the need of administering antibiotics to every patient who presents to the ED with AECOPD.

Design and method

This is a case-control observational study using retrospective chart review to obtain patients’ data from our hospital data warehouse. Data on patients with the primary diagnosis of AECOPD in the past two years were retrieved. A comparison between those who had VRP on arrival in the ED and those who did not have a VRP obtained was performed. We also compared ED throughput time for patients with AECOPD who received antibiotics to those who did not receive antibiotics. Only patients between the ages of 18 and 64 were included in the study. Patients with other preexisting health conditions such as cardiac diseases, neurological problems, and abdominal complaints were excluded. Patients who required hospitalization and pregnant patients were excluded from the study.

Results

We collected the data of 340 patients who met the study criteria. Of the 340 patients enrolled, 65 (19%) received the VRP test and 275 (81%) did not receive VRP test. Among the 65 patients who received the VRP test, 45 (70%) had a virus etiology detected and reported in the ED (p=0.001). Also, 138 (50.2%) did not receive VRP test and were not given antibiotics, and 137 (49.8%) did not receive VRP test but were treated with antibiotics; 11 patients received antibiotics despite haven tested positive to a virus. The result showed that those who received antibiotics with no VRP test on arrival in the ED had a shorter throughput time compared to patients who did not receive antibiotics but received VRP test.

Conclusion

The study is a quality improvement study to help determine the efficacy and appropriateness of ordering a VRP prior to ED disposition and the impact of overall ED throughput time for each patient presenting with AECOPD. The study showed that antibiotics did play a significant role in the duration of the throughput time in patients with AECOPD. However, rapid VRP testing was indeed associated with a trend toward decreased antibiotic use in the ED.

## Introduction

Chronic obstructive pulmonary disease (COPD) is defined as a chronic airflow limitation secondary to airways abnormalities most commonly secondary to noxious chemical gaseous/particle exposure such as cigarette use. A COPD exacerbation can be caused by pathogens such as viruses or bacteria or could be triggered by environmental factors such as exposure to smoke or air pollution and smoking habits.

The Global Initiative for Chronic Obstructive Lung Disease (GOLD) defines an acute exacerbation of COPD as “an acute event characterized by worsening of the patient’s respiratory symptoms that is beyond normal day-to-day variations and leads to a change in medication” [1]. This is typically further refined to contain three hallmark symptoms of an increase in cough frequency and/severity, resulting in dyspnea, complicated by their preexisting lung disease.

COPD exacerbations has been a thoroughly studied topic due to the associated burden on the health care delivery system. Multiple investigations have focused on antibiotic stewardship including the use of viral respiratory panels (VRPs) with the intent to reduce unnecessary antibiotic prescriptions [2]. Other studies including C-reactive protein (CRP) and pro-calcitonin testing [3,4] have tested pro-calcitonin levels in acute exacerbation chronic obstructive pulmonary disease (AECOPD) patients for a possible viral etiology and proposed that the elevation of pro-calcitonin or CRP in the patient’s blood sample has a strong association with a bacterial source. Given the volume of patients presenting to the Emergency Department (ED), efficient and appropriate medical treatment is important. Patients are typically given a broad-spectrum antibiotic such as azithromycin, doxycycline, cefuroxime, levofloxacin, or Bactrim. As with all medication, there is a risk versus benefit, which must be weighed upon prescribing. In patients with an unknown source of exacerbation, they are typically given an antibiotic prescription that covers a possible bacterial cause of the AECOPD along with a steroid prescription that reduces airway inflammation in their lungs [5]. An area of concern is for patients who receive an antibiotic prescription with an identified viral source in the absence of any reason to suspect a bacterial infection. This now predisposes the patient to the financial burden of paying for the antibiotic, the microbial risk of developing bacterial resistance to misused antibiotics, and the potential for side effects and drug-drug interactions with their daily medications [6].

One must also consider the use of hospital resources necessary to obtain and process a VRP. This involves nursing staff, lab technicians, and an ample amount of time, which all affect overall healthcare costs and time the patient spends in the ED. A viral respiratory sample is taken from the patient’s nose, typically on a swab. The swab is sent to the lab in the hospital where the extraneous components such as nasal mucous are tested for potential viruses. The material is processed to detect the most common viruses, which comprise a list of well-studied viruses whose makeup has been thoroughly investigated in medical research.

## Materials and methods

The statistical program SAS (SAS Institute Inc., Cary, NC) was used for data analysis. Continuous data (overall ED length of stay/throughput time) were reported as medians and interquartile ranges, and categorical variables were reported as frequencies and percentages. Comparisons of overall ED LOS/throughput time between those receiving a VRP and those not receiving a VRP were reported using t-tests for continuous data. Patient data were obtained through a secure electronic health record (HER) system that required log-in. All identifying patient information was kept in the Principal Investigator’s office, behind a locked door. Electronic data were stored on a password-protected computer in a password-protected file.

During data collection, an individualized chart review was performed by the study investigator(s) and research study coordinator/associate to limit the amount of sensitive patient information reviewed. All information containing patients’ protected health information (PHI) were not included in the study. Data of more than 500 patients who met the inclusion criteria were reviewed from August 1, 2019, to June 30, 2021, focusing on multiple metrics, as explained above.

Two-tailed t-test analysis was used to determine the statistical significance of the correlation between VRP and antibiotics use with ED throughput time.

## Results

**Figure 1:**
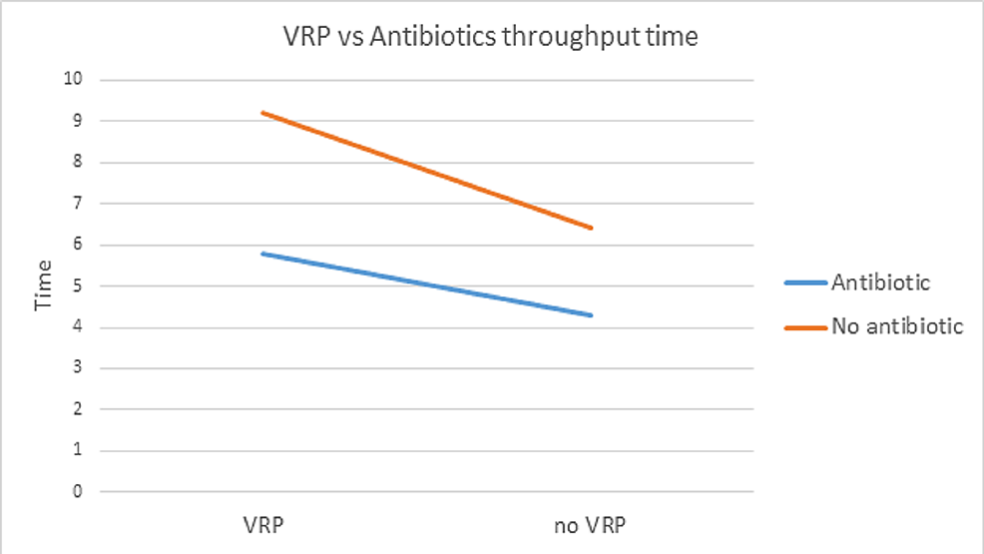
Comparing ED throughput time between administering VRP test upon arrival and antibiotics VRP, viral respiratory panel; ED, Emergency Department

The study reviewed ED visits among patients with the diagnosis of AECOPD beginning August 1, 2019, to June 30, 2021. A total of 340 patients met the criteria; 179 patients who received antibiotics had a mean ED throughput time of 5 hours with a standard deviation of 3.5, whereas 161 patients who did not receive antibiotics had a mean ED throughput time of 7 hours, with a standard deviation of 9.9 (Figure 1).

The use of VRP test in determining which patient requires antibiotics and which does not did not prove any change in patients’ overall outcome, but it did prolong patients’ ED stay due to the result wait-time. Antibiotic treatment rate occurred at an overall estimated rate of 52.6% (Table 1).

**Table 1:** Patient demographic depicting throughput time for those who received VRP test and patients who did not receive the VRP test with or without antibiotics prescription VRP, viral respiratory panel test; ED, Emergency Department

Variable	VRP test	No VRP test	Mean ED throughput time	Standard deviation	Standard error mean
Antibiotics	42	137	4.620	3.491	0.261
No antibiotics	23	138	6.798	9.888	0.779
Male	23	110	3.675	1.560	0.312
Female	42	165	4.529	2.787	0.199

ED management of AECOPD is shown in Table 2, which demonstrates both the investigations that were conducted to exclude other causes for the patients’ presenting symptoms and the treatment provided. Chest X-ray was the most common test that was performed on all patients upon arrival to the ED (100%); 249 (73.2%) patients had workup for possible cardiovascular origins for dyspnea and 80% had an infectious disease workup. A total of 328 (96.47%) patients received bronchodilators, 53% received antibiotics, and 79% received steroids inclusively (Table 2).

**Table 2:** Emergency Department management of AECOPD CT, computed tomography; ECG, electrocardiogram; BNP, brain natriuretic protein; CBC, complete blood count; CRP, C-reactive protein; AECOPD, acute exacerbations of chronic obstructive pulmonary disease

Patient assessment	Number of patients	Percentage (%)
Chest X-ray	340	100
Chest CT	210	61.76
ECG, Troponin, BNP	249	73.23
D-dimer	135	39.70
CBC	294	86.47
CRP	120	35.29
Blood culture	131	38.53
Sputum culture	129	37.94
Spirometry	136	40
Blood gas analysis	300	88.23
Received bronchodilator	328	96.47
Received antibiotics	179	52.64
Received steroid	268	78.82
Medical referral to pulmonologist	265	77.94

Interestingly, emergency room physicians preferred administering antibiotics to most patients with AECOPD and that seemed to decrease the throughput time in ED compared to administering VRP test. Reason could be the prolonged wait period to receiving the VRP test results, which further delays commencement of treatment in these patients. Also, the high volume of patients in the ED does play a role in the reason why ED physicians do not consider the test.

## Discussion

There are no clear benefits of antibiotics in patients diagnosed with AECOPD. Data from a national sample of ED visits were compared to our study and found that the overall rate of antibiotic use was 38%, which was similar to our use of antibiotics across our study period. Several meta-analyses studies have shown that the major determinant of recurrence in all GOLD stages of COPD severity was a history of exacerbations. The studies also suggest that frequent exacerbations is commonly seen in cases of moderate-to-severe stages of the disease [7].

In the multivariate analysis of data for the entire study period, in patients with AECOPD diagnosis in addition to association with previous exacerbations and disease severity, recurrence of exacerbation was seen especially in patients with other comorbidities such as gastroesophageal reflux disease (GERD) and obesity. Gender was associated with exacerbation frequency in our study, with 207 (61%) females; however, it was confounded with other variables such as smoking history, history of COPD, and obesity. Our study also suggests that chronic bronchitis was not associated with exacerbations despite previous reports that cough and sputum production are interrelated to AECOPD [8-10]. Although our study showed that 42 (64.6%) patients who received VRP test were also prescribed antibiotics, it can be explained due to the fact that viral respiratory infections may, in many cases, increase the risk of bacterial co-infections in COPD patients and that a positive VRP test does not completely rule out a concomitant bacterial infection. Our study further suggests that in carefully selected patients (patients with negative bacterial sputum cultures, cardiac disease patients, pregnant patients, and patients whose chest X-ray showed no infiltrates), administering VRP test may change antibiotic prescription pattern at discharge.

Several studies have tried to identify patients with AECOPD who are most likely to benefit from antibiotic therapy and patients who may not. While prescribing antibiotics to patients with severe COPD exacerbations requiring ICU level of care is recommended, there are no clear guidelines addressing patients with less severe cases [11]. Based on these criteria, guidelines recommended by GOLD for the use of antibiotics in patients who presents with worsening dyspnea, purulent sputum production, and large volume of sputum expectorate [12] are still being applied in our ED. Similarly, the Canadian Thoracic Society guidelines recommend antibiotics use only in more severely ill patients with AECOPD with increased purulent sputum production and dyspnea in the outpatient setting [13]. However, not all patients with AECOPD present in the ED with purulent sputum production or with positive bacteria sputum test. Thus, with the rise in antibiotics administration in patients with AECOPD and with increased antimicrobial resistance, it is crucial that we strive to derive more effective methods of identifying biochemical or microbiological markers for non-bacterial source of AECOPD, which will help direct the use of antibiotics for its right purposes.

Limitations

Our study only focused on one hospital center, and the patient sample does not represent the entire population. Data were collected cumulatively over a period of two years and during the peak of the (COVID-19) pandemic, and therefore not all COPD patients were correctly identified due to the influx of patients with severe COVID-19 symptoms.

## Conclusions

Patients presenting with a diagnosis of AECOPD can very much benefit from the use of VRP test. This test can significantly decrease the rate of antibiotics prescription in cases of AECOPD when administered to this group of patients upon arrival in the ED. Although the wait-time for the results could pose a daunting task and delay in treatment for patients, we strongly believe that the use of this test will allow for more targeted use of antibiotics in patients presenting to the ED with mild-to-moderate COPD exacerbations, thereby eliminating the negative impact of antimicrobial resistance. There is still a need for further studies that could elaborate more on the efficacy of VRP test practices on patient outcomes and antimicrobial resistance in the practice of medicine.
